# The risk of coronary artery disease in patients with rheumatoid arthritis using Chinese herbal products and conventional medicine in parallel: a population-based cohort study

**DOI:** 10.1186/s12906-020-02894-8

**Published:** 2020-03-30

**Authors:** Han-Hua Yu, Nai-Huan Hsiung, Jen-Huai Chiang, Hsuan-Shu Shen

**Affiliations:** 1 Division of Rheumatology, Allergy and Immunology, Chang Gung Memorial Hospital, Linkou, Taoyuan, Taiwan; 2 Department of Chinese Medicine, Hualien Tzu Chi Hospital, Buddhist Tzu Chi Medical Foundation, No. 707, Sec. 3, Chung-Yang Rd., Hualien, Taiwan 970; 3 Department of Nursing, Asia University, Taichung, Taiwan; 4 Management Office for Health Data, China Medical University Hospital, Taichung, Taiwan; 5 College of Medicine, China Medical University, Taichung, Taiwan; 6 School of Post-Baccalaureate Chinese Medicine, Tzu Chi University, Hualien, Taiwan

**Keywords:** Rheumatoid arthritis, Coronary artery disease, Chinese herbal products

## Abstract

**Background:**

Few studies have evaluated the association between the risk of coronary artery disease (CAD) and the use of Chinese herbal products (CHP) in patients with rheumatoid arthritis (RA). This study investigated the risk of CAD among patients with RA using CHP in combination with conventional medicine.

**Methods:**

A retrospective cohort study was conducted using the Taiwan National Insurance Research Database to assess 22,353 patients who had been newly diagnosed with RA between 1997 and 2010. Patients were assigned to the CHP group or non-CHP group according to their use or nonuse of CHP after being diagnosed with RA. The Cox proportional hazards model was used to estimate the hazard ratio (HR) of CAD for a 1:1 matched sample.

**Results:**

Both the CHP and non-CHP groups comprised 4889 patients after 1:1 matching. The risk of CAD was significantly reduced in the CHP group [adjusted HR (aHR): 0.59, 95% confidence interval (CI): 0.50–0.71] compard with the non-CHP group. Those who used CHP for > 180 days had an even lower risk of CAD than users with CHP usage less than 30 days (aHR: 0.64, 95% CI: 0.43–0.95). Additionally, frequently prescribed formulae, such as Kuei-Chih-Shao-Yao-Chih-Mu-Tang, Tang-Kuei-Nien-Tung-Tang, and Shu-Ching-Huo-Hsieh-Tang, were associated with a reduced risk of CAD.

**Conclusion:**

The use of CHP was associated with a lower risk of CAD in patients with RA. Additional randomized controlled trials are required to assess any causal relationship between the effect of CHP usage and the risk of CAD.

## Background

The risk of coronary artery disease (CAD) is known to be elevated in patients with rheumatoid arthritis (RA). A retrospective cohort study found a 39–58% increased risk of major cardiovascular events in patients with RA in comparison with the general population [1], and a meta-analysis of observational studies found that the risk of cardiovascular events was 48% higher in patients with RA [2]. One possible reason for this is the systemic chronic inflammation in patients with RA contributing to the pathway of atherosclerosis, from plaque formation to the rupture of plaque and thrombosis [3, 4]. Furthermore, in the development of both RA and atherosclerosis, the same proinflammatory cytokines, i.e., interleukin (IL)-6 and tumor necrosis factor-alpha (TNF-α), are at work [3]. The release of IL-6 and TNF-α is stimulated by chronic systemic inflammation, and these proinflammatory cytokines which increase vascular calcification and accelerate atherosclerosis progression, from early atheroma formation to thrombus development, may play a primary role in increasing the risk of CAD in patients with RA [5–7].

The conventional treatment for RA includes nonsteroidal anti-inflammatory drugs (NSAIDs), corticosteroids, disease-modifying antirheumatic drugs (DMARDs), and TNF antagonists. These commonly used drugs have an anti-inflammatory mechanism. Typically, the combined use of NSAIDs and DMARDs is effective for treating patients with RA. However, certain NSAIDs and corticosteroids are known to increase the risk of CAD. A meta-analysis found that the risk of cardiovascular events increased by 18 and 47% in patients with RA who were taking NSAIDs and corticosteroids, respectively [8]. Conversely, DMARDs have been found to have a protective effect regarding CAD. A previous report indicated that DMARDs, particularly methotrexate, were associated with an 18% reduction in the risk of CAD [9]. Apart from its anti-inflammatory effect, methotrexate has some adverse effects, such as nausea, vomiting, hepatotoxicity, nephrotoxicity, and myelosuppression [10]. Subesinghe and Scott (2015) revealed that the most common reason for the permanent discontinuation of methotrexate was its adverse effects [11]. Some patients discontinued the conventional treatment because due to its adverse effects, which could induce disease reactivation. In contrast, most of complementary and alternative medicine (CAM) was associated with only minor adverse effects [12]. Accordingly, patients have tended to seek CAM for pain relief or symptom management in recent times [13].

Patients with RA were observed increasingly to use both CAM and conventional medicine in parallel. In the United States, a population-based survey found that 41% of patients with RA used some kind of CAM [14]. Eighty-two percent of RA patients reportedly use CAM in Korea, and traditional Asian medicine is the most widespread type of treatment [15]. In Taiwan, nearly 30% of RA patients have been reported to use Chinese herbal products (CHP), and certain herbs in commonly prescribed formulae, such as *Paeonia lactiflora* Pall. (Shao-Yao) and *Platycladus orientalis* (Linn.) (Huang-Po), are known to have an anti-inflammatory effect [16, 17]. Kuei-Chih-Shao-Yao-Chih-Mu-Tang, the formula used in clinical practice for RA, has shown promising anti-inflammatory effects in lowering the erythrocyte sedimentation rate (ESR) and alleviating morning stiffness [18]. *Paeonia lactiflora* Pall. (Shao-Yao), in addition to its anti-inflammatory properties, has also been implicated in the prevention of hepatotoxicity in concurrent methotrexate use. A randomized controlled trial found that patients with RA who received total glucosides of peony in combination with leflunomide and methotrexate had lower rates of hepatotoxicity [19, 20]. However, despite the promising anti-inflammatory effect of CHP reported in previous studies, the association between the effect of CHP and risk of CAD remains unknown. Thus, this present study aimed to investigate the risk of CAD in patients with RA who were using CHP and conventional medicine in parallel.

## Methods

### Data source

This study used reimbursement claims data from the Taiwan National Health Insurance (NHI) program implemented in March 1995. The NHI program is a single-payer, compulsory universal health insurance program that has offered comprehensive medical care coverage to 99% of the Taiwanese population and has contracts with 97% of the country’s hospitals and clinics (http://www.nhi.gov.tw/english/index.aspx). The National Health Insurance Research Database (NHIRD) has documentation regarding every medical treatment reimbursed by the NHI program, including traditional Chinese medicine treatments.

The data sets of the study comprised registry information for beneficiaries, ambulatory and inpatient care claims, and the Registry for Catastrophic Illness of NHIRD. We used ambulatory and inpatient care records of patients between 1997 and 2010 from the Registry for Catastrophic Illness to identify study subjects for follow-up until the end of 2011. As previously described, RA is statutorily included in the catastrophic illness category with solid evaluation criteria [21]. The ambulatory care claims record contain an individual’s sex, date of birth, visit dates, and International Classification of Diseases, Ninth Revision, Clinical Modification (ICD-9-CM) codes for three primary diagnoses. Inpatient claims contain ICD-9-CM codes for principal diagnosis and up to four secondary diagnoses. The ICD-9-CM codes include physicians who practice Chinese medicine. According to the principle of data protection of NHIRD, any data in the NHIRD that could be used to identify patients or care providers, including medical institutions and physicians, are scrambled before being sent to the National Health Research Institutes for database construction and then further scrambled before being released to each researcher. Theoretically, it is impossible to query the data in isolation alone to identify individuals at any level by using this database. The requirement regarding informed consent in the present study was waived. This study was approved by the Institutional Review Board of the China Medical University (CMUH104-REC2–115).

### Study population selection

**Fig. 1:**
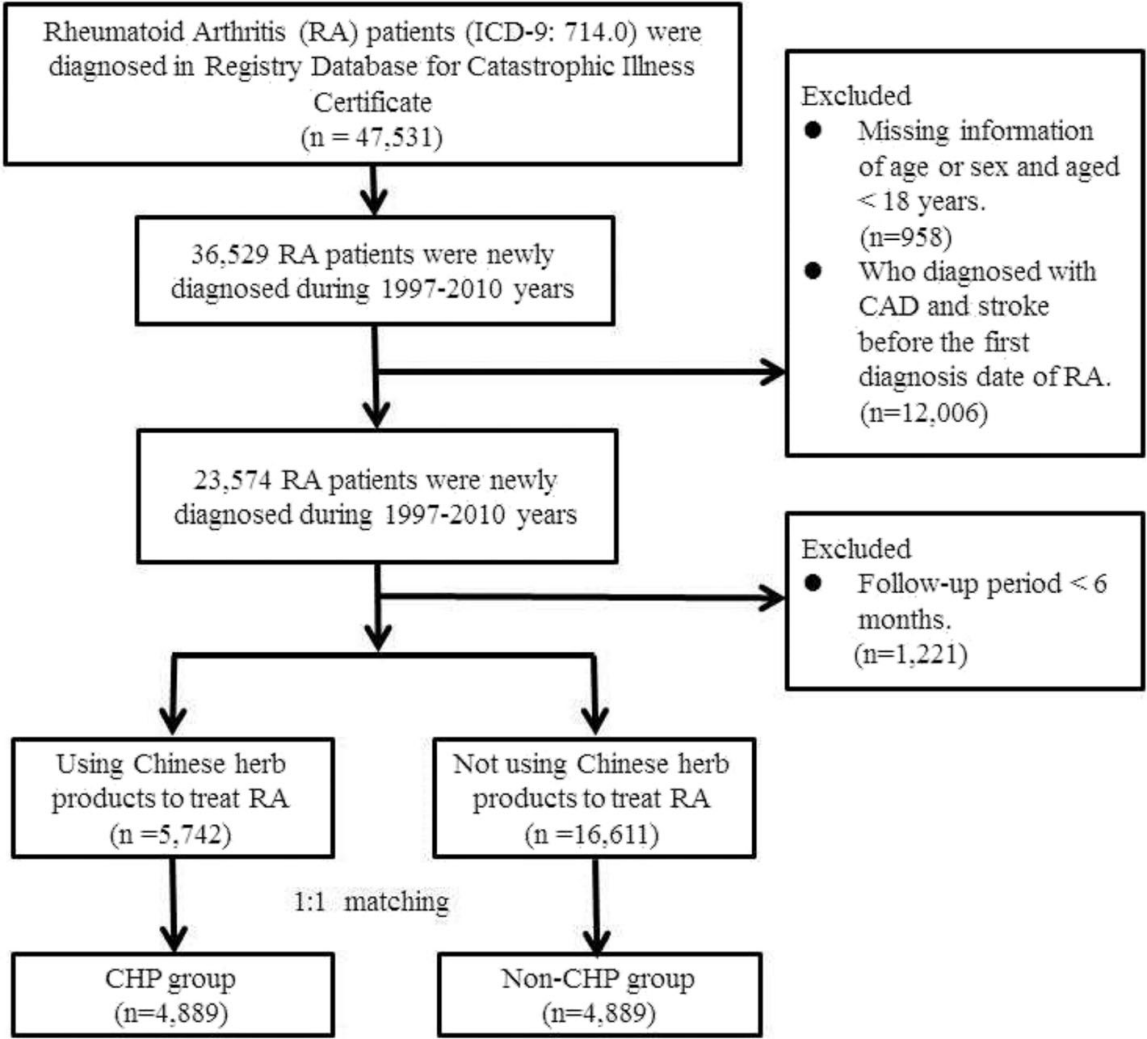
Flow recruitment chart of patients with rheumatoid arthritis (RA) from the Registry Database for Catastrophic Illness Certificate. After excluding patients not fitting the inclusion criteria, both the CHP and non-CHP groups comprised 4889 patients after 1:1 matching by sex, age, index year, and initial diagnosis year of RA

This was a retrospective cohort study, in which the population was followed up between January 1, 1997 and December 31, 2011. Patients diagnosed with RA (ICD-9-CM code 714.0) between January 1, 1997 and December 31, 2010 formed our study cohort and were followed up until December 31, 2011. Patients aged < 18 years and those who had either withdrawn from insurance coverage or been diagnosed with CAD (ICD-9-CM code 410–414) and stroke (ICD-9-CM 430–438) before the first diagnosis date of RA were excluded from the study, including patients whose follow-up period was < 6 months. In total, 22,353 patients with RA during the period 1997–2010 were identified (Fig. 1).

The participants use of CHP, in parallel with their conventional treatments from the date of their initial RA diagnosis, were allocated to the CHP group. Those RA patients who were using conventional medicine without receiving any CHP were assigned to the non-CHP group. The index date for the non-CHP group was randomly selected between the initial RA diagnosis date and the study’s endpoint. The date on which CHP was first used to treat RA was used as the index date for the CHP group. Prior to the matching, there were 5742 patients in the CHP group and 16,611 patients in the non-CHP group. A 1:1 frequency matching was applied for sex, age (in 5-year increments), index year, and initial RA diagnosis year. Following the frequency matching, both the CHP and non-CHP groups comprised 4889 patients with RA.

### Primary outcome

The primary outcome was CAD (ICD-9-CM codes 410–414) diagnosed by a cardiologist during the 15-year follow-up period with at least three outpatient claims or one hospital claim. All eligible patients were followed up from the index date to the date of diagnosis of CAD, withdrawal from the NHI program, or the study endpoint (December 31, 2011), whichever occurred first.

### Characteristics, comorbidities, and drugs

The sociodemographic variables included age and sex. Comorbidities were identified using ICD-9-CM codes from the database of outpatient and inpatient visits. The baseline comorbidities were defined as disease occurring within 1 year before the index date, with at least three associated outpatient claims or at least one hospital claim. Baseline comorbidities included diabetes mellitus (ICD-9-CM code: 250), hypertension (ICD-9-CM codes: 401–405), hyperlipidemia (ICD-9-CM code: 272), chronic obstructive pulmonary disease (ICD-9-CM codes: 491–496), and end-stage renal disease (ICD-9-CM code: 585). We also considered medication use, including NSAIDs, corticosteroids, DMARDs and TNF antagonists from the initial RA diagnosis date to the study endpoint.

### Statistical analysis

The continuous variables were reported as the mean and standard deviation, whereas the categorical variables were reported as numbers and percentages. Differences in the proportions and means were evaluated using the chi-square test or *t*-test. The Cox proportional hazards model was used to estimate the hazard ratio (HR) of CAD after the use of CHP, controlling for potential confounding factors including age, sex, diabetes mellitus, hypertension, hyperlipidemia, COPD, ESRD and RA related medicine usage. For categorical covariates, the Kaplan–Meier and log-rank tests were performed. A *P* value < 0.05 was considered statistically significant. In this study, SAS, version 9.4 (SAS Institute Inc., Cary, NC, USA), was used for the statistical analysis.

## Results

Following the 1:1 matching, there were more than six times more females than male patients in both groups. Regarding in the aspect of comorbidities, the proportion of hypertension was significantly lower in the CHP group than in the non-CHP group but, by contrast, the proportion of COPD was higher in the CHP group than in the non-CHP group. Moreover, the proportion of DMARDs and TNF-antagonist use in the CHP group was significantly higher than that in the non-CHP group. The mean (median) follow-up period was 5.38 (4.94) years and 4.88 (4.34) years for the CHP group and non-CHP group, respectively (Table 1).

**Table 1:** Characteristics of patients with rheumatoid arthritis classified according to the use of Chinese herbal products

Variable	Patients with Rheumatoid Arthritis				*p*-value*
	Non-CHP group (*n* = 4889)		CHP group (*n* = 4889)		
	n	%	n	%	
**Sex**					0.99
Women	4226	86.44	4226	86.44	
Men	663	13.56	663	13.56	
**Age group**					0.99
18–39	1260	25.77	1260	25.77	
40–59	2948	60.3	2948	60.3	
≥ 60	681	13.93	681	13.93	
Mean ± SD (years)^a^	47.51 (11.55)		47.43 (11.56)		0.3695
**Baseline Comorbidity**					
Diabetes mellitus	455	9.31	404	8.26	0.0685
Hypertension	1165	23.83	984	20.13	<.0001
Hyperlipidemia	606	12.4	622	12.72	0.6254
COPD	742	15.18	897	18.35	<.0001
ESRD	59	1.21	41	0.84	0.0704
**Drug used**					
NSAID	4863	99.47	4876	99.73	0.037
Corticosteroid	226	4.62	246	5.03	0.3454
DMARD	4585	93.78	4780	97.77	<.0001
TNF-antagonists	713	14.58	1161	23.75	<.0001
**Duration between diagnosed date and index, years (mean, median)**^**a**^	2.58 (1.67)		2.53 (1.63)		0.3905

*chi-square test; ^a^*t*-test

Abbreviations: *CHP* Chinese herbal products; *SD* standard deviation; *COPD* chronic obstructive pulmonary disease; *ESRD* end-stage renal disease

The mean (median) of the follow-up period was 5.38 (4.94) years for the CHP group and 4.88 (4.34) years for the non-CHP group

**Fig. 2:**
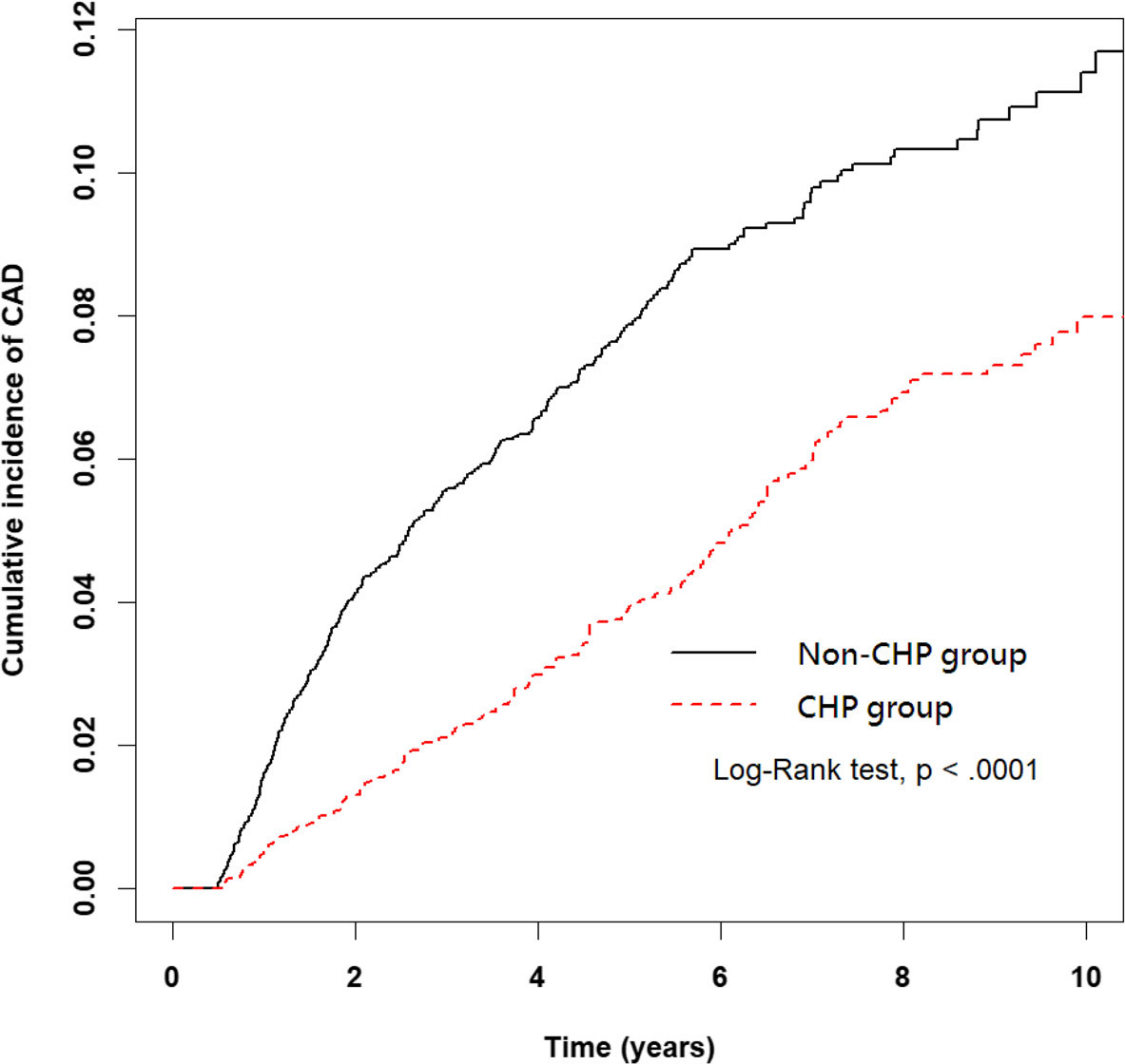
Cumulative incidence of CAD between the CHP group and the non-CHP group. The cumulative incidence of CAD in the CHP group is significantly lower than in the non-CHP group (log-rank test, *p* < .0001). Abbreviations: CAD, coronary artery disease. CHP, Chinese herbal products

A total of 568 CAD cases were identified during the follow-up period, with an incidence rate of CAD of 8.06 per 1000 person-years in the CHP group and 14.93 per 1000 person-years in the non-CHP group. Univariate and multivariate Cox proportional hazards models were estimated according to the risk of CAD using HR and a 95% confidence interval (CI) in the cohort of patients with RA in the CHP and non-CHP groups. The Kaplan-Meier analysis demonstrated that the cumulative incidence of CAD was significantly lower in the CHP group than in the non-CHP group (log-rank test, *p* < 0.0001) (Fig. 2). Patients in the CHP group were more likely to have a decreased risk of CAD (crude HR: 0.55, 95% CI: 0.46–0.65) compared with the non-CHP group. Following the multivariate adjustment, the HR remained the same (aHR: 0.59, 95% CI: 0.5–0.71) (Table 2).

**Table 2:** Incidence rates, hazard ratios, and confidence intervals of coronary artery disease in patients with rheumatoid arthritis using and not using Chinese herbal products, stratified by sex, age, comorbidities, and drug used

Variables	Rheumatoid Arthritis						Compared with non-CHP group	
	Non-CHP group (*n* = 4889)			CHP group (*n* = 4889)			Crude HR	Adjusted HR
	Event	Person years	IR^**†**^	Event	Person years	IR	(95% CI)	(95% CI)
**Total**	356	23,839	14.93	212	26,309	8.06	0.55 (0.46–0.65)***	0.59 (0.5–0.71)***
**Sex**								
Women	294	20,892	14.07	172	22,844	7.53	0.54 (0.45–0.65)***	0.59 (0.49–0.72)***
Men	62	2947	21.04	40	3465	11.54	0.55 (0.37–0.82)**	0.55 (0.36–0.85)**
**Age group**								
18–39	35	6580	5.32	25	6942	3.60	0.68 (0.41–1.13)	0.77 (0.45–1.32)
40–59	225	14,471	15.55	125	15,966	7.83	0.51 (0.41–0.63)***	0.57 (0.46–0.72)***
≥ 60	96	2789	34.43	62	3401	18.23	0.55 (0.4–0.76)***	0.6 (0.43–0.84)**
**Baseline Comorbidity**								
Diabetes mellitus								
No	298	21,861	13.63	189	24,227	7.80	0.58 (0.48–0.69)***	0.61 (0.51–0.74)***
Yes	58	1978	29.32	23	2081	11.05	0.39 (0.24–0.63)***	0.49 (0.29–0.82)**
Hypertension								
No	194	18,851	10.29	130	21,509	6.04	0.59 (0.47–0.74)***	0.6 (0.48–0.76)***
Yes	162	4989	32.47	82	4800	17.08	0.54 (0.41–0.7)***	0.6 (0.45–0.78)***
Hyperlipidemia								
No	294	21,399	13.74	178	23,381	7.61	0.56 (0.46–0.67)***	0.6 (0.5–0.73)***
Yes	62	2440	25.41	34	2928	11.61	0.47 (0.31–0.71)***	0.53 (0.34–0.81)**
COPD								
No	269	20,480	13.13	154	21,839	7.05	0.54 (0.45–0.66)***	0.62 (0.51–0.77)***
Yes	87	3359	25.90	58	4470	12.98	0.5 (0.36–0.7)***	0.51 (0.36–0.72)***
ESRD								
No	346	23,608	14.66	207	26,109	7.93	0.55 (0.46–0.65)***	0.59 (0.49–0.71)***
Yes	10	231	43.27	5	199	25.10	0.56 (0.19–1.65)	1.05 (0.22–4.93)
**Drug used**								
NSAID								
No	2	70	28.66	0	40	0.00	–	–
Yes	354	23,770	14.89	212	26,269	8.07	0.55 (0.46–0.65)***	0.59 (0.5–0.71)***
Corticosteroid								
No	340	22,629	15.02	199	24,834	8.01	0.54 (0.45–0.64)***	0.58 (0.49–0.7)***
Yes	16	1210	13.22	13	1475	8.82	0.66 (0.32–1.38)	0.78 (0.36–1.69)
DMARD								
No	41	1349	30.39	12	611	19.65	0.71 (0.37–1.34)	0.71 (0.35–1.42)
Yes	315	22,490	14.01	200	25,698	7.78	0.56 (0.47–0.67)***	0.58 (0.48–0.7)***
TNF-antagonists								
No	339	19,953	16.99	189	19,768	9.56	0.57 (0.48–0.68)***	0.58 (0.49–0.7)***
Yes	17	3886	4.37	23	6541	3.52	0.79 (0.42–1.48)	0.69 (0.36–1.32)

Abbreviations: *IR* incidence rates, per 1000 person-years; *HR* hazard ratio; *CI* confidence interval; *CHP* Chinese herbal products; *COPD* chronic obstructive pulmonary disease; *ESRD* end-stage renal disease; *NSAID* nonsteroidal anti-inflammatory drugs; *DMARDs* disease-modifying antirheumatic drugs; *TNF-antagonists* tumor necrosis factor-antagonists

Adjusted HR: adjusted for CHP use, age, sex, diabetes mellitus, hypertension, hyperlipidemia, COPD, ESRD, NSAID use, corticosteroid, DMARD and TNF-antagonists in Cox proportional hazards regression

**p < 0.01; *** *p* < 0.001

Stratified by sex, the incidence rate of CAD in the CHP group was 7.53 per 1000 person-years in women and 11.54 per 1000 person-years in men, whereas, in the non-CHP group, it was 14.07 per 1000 person-years in women and 21.04 per 1000 person-years in men. The risk of CAD was significantly lower among women (0.59-fold, 95% CI: 0.49–0.72) and men (aHR: 0.55, 95% CI: 0.36–0.85) in the CHP group compared the non-CHP group. In the 40–59 and > 60-year age groups, the risk of CAD was lower in the CHP group than in the non-CHP group (Table 2). Patients with RA who received CHP treatment were found to have a significantly lower adjusted HR regarding the risk of CAD (Table 3). In the CHP group, patients who used CHP for > 180 days had a lower risk of CAD than those who used CHP for < 30 days (aHR: 0.64, 95% CI: 0.43–0.95).

**Table 3:** Hazard ratios and 95% confidence intervals of coronary artery disease risk associated with cumulative number of days of CHP use among patients with rheumatoid arthritis

	n	Event	Hazard Ratio(95% CI)		Hazard Ratio(95% CI)	
		no.	Crude	Adjusted^**†**^	Crude	Adjusted^**†**^
**Non-CHP group**	4889	356	1(reference)	1(reference)	–	–
**CHP group**	4889	212	0.55 (0.46–0.65)***	0.59 (0.5–0.71)***		
< 30 days	2280	99	0.58 (0.46–0.72)***	0.62 (0.50–0.78)***	1 (reference)	1 (reference)
30–180 days	1675	80	0.59 (0.47–0.76)***	0.67 (0.52–0.85)**	1.01 (0.75–1.36)	1.05 (0.78–1.41)
> 180 days	934	33	0.40 (0.28–0.58)***	0.41 (0.29–0.59)***	0.67 (0.45–1.00)	0.64 (0.43–0.95)*

Crude HR^*^ represented relative hazard ratio

Adjusted HR^†^ represented adjusted hazard ratio: mutually adjusted for age, sex, diabetes mellitus, hypertension, hyperlipidemia, COPD, ESRD, NSAID use, corticosteroid, DMARDs, and TNF-antagonists in Cox proportional hazards regression

Abbreviations: *HR* hazard ratio; *CI* confidence interval; *CHP* Chinese herbal products; *COPD* chronic obstructive pulmonary disease; *ESRD* end-stage renal disease

*p < 0.05, **p < 0.01, ***p < 0.001

The three most commonly prescribed herbs were *Corydalis ambigua* Cham. & Schltdl. (Yen-Hu-So), *Spatholobus suberectus* Dunn (Chi-Hsieh- Teng), and *Morus alba* L. (Sang-Chih), and the three most commonly prescribed formulae were Kuei-Chih-Shao-Yao-Chih-Mu-Tang, Tang-Kuei-Nien-Tung-Tang, and Shu-Ching-Huo-Hsieh-Tang. The Cox proportional hazards models showed that Kuei-Chih-Shao-Yao-Chih-Mu-Tang, Tang-Kuei-Nien-Tung-Tang, Shu-Ching-Huo-Hsieh-Tang, Tu-Huo-Chi-Sheng-Tang, and Chia-Wei-Hsiao-Yao-San were associated with a reduced risk of CAD. (Table 4).

**Table 4:** Hazard Ratios and 95% confidence intervals of coronary artery disease (CAD) risk associated with type of herbal formulae among RA patients

CHP prescription	CAD		Hazard Ratio(95% CI)	
	n	No. of Event	Crude	Adjusted^**†**^
**Non-CHP group**	4889	356	1 (reference)	1 (reference)
**CHP group**				
**Single Herb**				
1. *Corydalis ambigua* Cham. & Schltdl, family Papaveraceae (Yen-Hu-So, 延胡索)	1099	35	0.39 (0.28–0.55)***	0.45 (0.31–0.64)***
2. *Spatholobus suberectus* Dunn, family Leguminosae (Chi-Hsieh-Teng, 雞血藤)	935	36	0.46 (0.33–0.65)***	0.53 (0.37–0.75)***
3. *Morus alba* L., family Moraceae (Sang-Chih, 桑枝)	844	33	0.47 (0.33–0.67)***	0.56 (0.39–0.80)**
4. *Aconitum wilsonii* Stapf ex Veitch., family Ranunculaceae (Pao-Fu-Tzu, 炮附子)	764	25	0.42 (0.28–0.63)***	0.52 (0.35–0.79)**
5. *Salvia miltiorrhiza* Bunge, family Lamiaceae (Tan-Tsen, 丹參)	721	29	0.48 (0.33–0.70)***	0.50 (0.34–0.73)***
**Formulae**				
1. Kuei-Chih-Shao-Yao-Chih-Mu-Tang	1726	70	0.49 (0.38–0.63)***	0.55 (0.43–0.72)***
2. Tang-Kuei-Nien-Tung-Tang	1390	56	0.48 (0.36–0.64)***	0.54 (0.40–0.72)***
3. Shu-Ching-Huo-Hsieh-Tang	1165	40	0.40 (0.29–0.56)***	0.43 (0.31–0.60)***
4. Tu-Huo-Chi-Sheng-Tang	816	25	0.35 (0.24–0.53)***	0.38 (0.25–0.57)***
5. Chia-Wei-Hsiao-Yao-San	720	19	0.32 (0.20–0.51)***	0.43 (0.27–0.69)***

Abbreviations: *IR* incidence rates, per 1000 person-years; *HR* hazard ratio; *CHP* Chinese herbal products; *COPD* chronic obstructive pulmonary disease; *ESRD* end-stage renal disease

Adjusted HR^†^: adjusted hazard ratio, mutually adjusted for accepted type of CHP, age, sex, diabetes mellitus, hypertension, hyperlipidemia, COPD, ESRD, NSAID uses, corticosteroid, DMARD and TNF- antagonists in Cox proportional hazard regression

**p < 0.01, ***p < 0.001

## Discussion

This population-based cohort study was performed as preparation for an evaluation of the association between CHP application and the risk of CAD in RA patients. We found that the risk of CAD in patients with RA who used CHP in combination with conventional treatments was 41% lower than that in patients who used conventional treatments only (aHR: 0.59, 95% CI: 0.5–0.71). Of the several commonly prescribed formulae, Kuei-Chih-Shao-Yao-Chih-Mu-Tang, Tang-Kuei-Nien-Tung-Tang, and Shu-Ching-Huo-Hsieh-Tang were associated with a lower risk of CAD. Moreover, patients using CHP for > 180 days had a 36% reduced risk of CAD compared with patients using CHP for < 30 days (aHR: 0.64, 95% CI: 0.43–0.95). The results of our study indicate a strong relationship between the use of CHP and a reduced risk of CAD in patients with RA. A possible explanation of this is that commonly prescribed CHP treatments might have anti-inflammatory effects [18–20]. The proinflammatory cytokines associated with RA, which is an autoimmune and chronic inflammatory disorder, are the same as those seen in the development of atherosclerosis, which is thought to be the main driver of CAD [3, 5, 6]. The use of CHP in combination with other therapies might block the inflammation process and thereby lower the risk of CAD.

In both groups, there were 6.5 times more women than men, and 85% of the patients were under the age of 60. According to previous research, the prevalence of RA is higher among woman than men, and the sex ratio is around 4.5:1 [22]. Moreover, women are more prone then men to use Chinese herbal medicine in Taiwan [23], which may explain the difference in the respective number of female and male participants in our study. Furthermore, we observed that the incidence rate of CAD in male patients is higher than that in female patients. One possible reason may be the effect of hormones. Compared with men, the incidence rate of CAD is significantly lower in premenopausal women because of the protective effect of endogenous estrogens [24]. The majority of the female patients are under 60-years old, which equates to the pre-menopause period. Estrogens are reported to reduce the atherosclerotic process and lower the effect of total cholesterol. The protective mechanisms account for the lower incidence rate of CAD in female patients compared to male patients with rheumatoid arthritis.

Some commonly prescribed herbs and herbal formulae have been found to lower the risk of CAD. One possible reason is that these frequently prescribed formulae and herbs may have anti-inflammatory effects. Kuei-Chih-Shao-Yao-Chih-Mu-Tang, which originated in Shang-Han-Lun and was the most commonly used formula in our study, can alleviate morning stiffness and lower the ESR, a nonspecific inflammatory factor [18]. The individual herbs contained in Kuei-Chih-Shao-Yao-Chih-Mu-Tang, such as *Paeonia lactiflora* Pall. (Shao-Yao) and *Anemarrhena asphodeloides* Bge (Chih-Mu), have also been found to have potential anti-inflammatory effects. Chen (2011) found that *Paeonia lactiflora* Pall. (Shao-Yao) suppressed TNF-α-induced chemokine production by blocking nuclear factor-κB and the extracellular signal-regulated kinase pathway in human dermal microvascular endothelial cells [25]. Moreover, Chen (2013) documented that *Paeonia lactiflora* Pall. (Shao-Yao) had anti-inflammatory effects by lowering the ESR and CRP levels and a protective effect against hepatotoxicity in a combination treatment regimen with leflunomide and methotrexate after 24 weeks of treatment [19]. *Anemarrhena asphodeloides* Bge (Chih-Mu) was found to have cardioprotective effects on isoproterenol-induced myocardial infarction in rats [26]. These results suggest that pretreatment with *Anemarrhena asphodeloides* Bge (Chih-Mu) extracts reversed the elevation of IL-6 and TNF-α induced by isoproterenol. Additionally, certain herbs in Tang-Kuei-Nien-Tung-Tang were also found to have a potential anti-inflammatory effect. Hang (2018) demonstrated that *Scutellaria baicalensis* Georgi (Huang-Chin) could lower CRP and blood lipids in patients with RA and CAD [27]. Other herbs, such as *Angelica sinensis* (Oliv.) Diels (Tang-Kuei) and *Sophora flavescens* Aiton (Ku-Tsen), were also found to have inhibitory effects on TNF-α in an animal study [28]. Thus, commonly used CHPs might suppress the production of IL-6 and TNF-α, the same proinflammatory cytokines active in the process of atherosclerosis, to reduce the risk of CAD.

In the CHP group, patients who had used CHP for > 180 days were found to have a lower risk of CAD than those who had used CHP for < 30 days. A possible explanation for this is that CHP requires at least 180 days to achieve a steady protective effect in patients with RA. The cumulative number of days of CHP use was similar to the treatment duration of methotrexate recommended by the European League Against Rheumatism [29]. Moreover, the results obtained from the minimum number of days of CHP use were consistent with those of previous randomized controlled trials. Patients with RA taking *Paeonia lactiflora* Pall. (Shao-Yao) extracts combined with treatment with leflunomide and methotrexate for 24 weeks had lower CRP levels and ESR [19]. Additionally, after 24 weeks of treatment with *Salvia miltiorrhiza* Bunge (Tan-Tsen) and *Pueraria lobata* (Willd.) Ohwi (Ko-Ken), there was an improvement in the carotid intima-media thickness in coronary patients [30]. RA is characterized by chronic systemic inflammation, and patients using CHP for > 180 days may achieve an improved anti-inflammatory effect that reduces the risk of CAD.

Finally, several important limitations of our study must be considered. First, certain factors, such as disease activity, disease severity, smoking, body mass index, diet, exercise habits, and stress levels, all of which are associated with CAD, were not measured and were unavailable from our database. However, previous study documented that the use of TNF-antagonists and DMARDs may decrease the risk of CAD [31, 32] Thus, we applied a Cox Proportional Hazards Model by controlling for the available potential confounding factors of CAD, including age, gender, comorbidities and medication, to make the two groups comparable. Second, there was a possibility of misclassification bias in the days of cumulative use of CHP. We presumed that all of the CHP prescribed was taken by the patients, but the actual dosage taken may have been overestimated due to noncompliance. Due to the impact of medication adherence, the protective effect of CHP against CAD may be underestimated. Third, the use of over-the-counter CHP was not recorded in the NHIRD. The database used in our study contained only records of the CHP prescribed by licensed physicians practicing traditional Chinese medicine; thus, the rate of CHP use may have been underestimated.

## Conclusion

This study revealed an association with decreased CAD risk in RA patients receiving CHP in combination with conventional treatments. In particular, RA patients who took CHP for > 180 days had a lower risk. Additionally, Kuei-Chih-Shao-Yao-Chih-Mu-Tang, Tang-Kuei-Nien-Tung-Tang, and Shu-Ching-Huo-Hsieh-Tang might be associated with a lower risk of CAD. Future randomized controlled trials are required to clarify the causal association between the use of CHP and the risk of CAD.

## Data Availability

The data that support the findings of this study are available from the Taiwan National Health Insurance Program but restrictions apply to the availability of these data, which were used under license for the current study, and so are not publicly available. Data are however available from the authors (Hsuan-Shu Shen) upon reasonable request and with permission of the Taiwan National Health Insurance Program.

## Abbreviations

aHR: Adjusted HR
CAD: Coronary artery disease
CAM: Complementary and alternative medicine
CHP: Chinese herbal products
CI: Confidence interval
CRP: C-reactive protein
DMARDs: Disease-modifying antirheumatic drugs
ESR: Erythrocyte sedimentation rate
HR: Hazard ratio
ICD-9-CM: International Classification of Diseases, Ninth Revision, Clinical Modification
IL: Interleukin
NHI: National Health Insurance
NHIRD: National Health Insurance Research Database
NSAIDs: Nonsteroidal anti-inflammatory drugs
RA: Rheumatoid arthritis
TNF-α: Tumor necrosis factor-alph

## References

[1] Ogdie A, Yu Y, Haynes K, Love TJ, Maliha S, Jiang Y, Troxel AB, Hennessy S, Kimmel SE, Margolis DJ (2015). Risk of major cardiovascular events in patients with psoriatic arthritis, psoriasis and rheumatoid arthritis: a population-based cohort study. Ann Rheum Dis.

[2] Avina-Zubieta JA, Thomas J, Sadatsafavi M, Lehman AJ, Lacaille D (2012). Risk of incident cardiovascular events in patients with rheumatoid arthritis: a meta-analysis of observational studies. Ann Rheum Dis.

[3] Libby P (2008). Role of inflammation in atherosclerosis associated with rheumatoid arthritis. Am J Med.

[4] Skeoch S, Bruce IN (2015). Atherosclerosis in rheumatoid arthritis: is it all about inflammation?. Nat Rev Rheumatol.

[5] Hartman J, Frishman WH (2014). Inflammation and atherosclerosis: a review of the role of interleukin-6 in the development of atherosclerosis and the potential for targeted drug therapy. Cardiol Rev.

[6] McKellar GE, McCarey DW, Sattar N, McInnes IB (2009). Role for TNF in atherosclerosis? Lessons from autoimmune disease. Nat Rev Cardiol.

[7] Lopez-Mejias R, Gonzalez-Gay MA (2019). IL-6: linking chronic inflammation and vascular calcification. Nat Rev Rheumatol.

[8] Roubille C, Richer V, Starnino T, McCourt C, McFarlane A, Fleming P, Siu S, Kraft J, Lynde C, Pope J (2015). The effects of tumour necrosis factor inhibitors, methotrexate, non-steroidal anti-inflammatory drugs and corticosteroids on cardiovascular events in rheumatoid arthritis, psoriasis and psoriatic arthritis: a systematic review and meta-analysis. Ann Rheum Dis.

[9] Micha R, Imamura F, Wyler von Ballmoos M, Solomon DH, Hernan MA, Ridker PM, Mozaffarian D (2011). Systematic review and meta-analysis of methotrexate use and risk of cardiovascular disease. Am J Cardiol.

[10] Salliot C, van der Heijde D (2009). Long-term safety of methotrexate monotherapy in patients with rheumatoid arthritis: a systematic literature research. Ann Rheum Dis.

[11] Subesinghe S, Scott IC (2015). Key findings from studies of methotrexate tapering and withdrawal in rheumatoid arthritis. Expert Rev Clin Pharmacol.

[12] Posadzki P, Watson LK, Ernst E (2013). Adverse effects of herbal medicines: an overview of systematic reviews. Clin Med (London, England).

[13] Efthimiou P, Kukar M (2010). Complementary and alternative medicine use in rheumatoid arthritis: proposed mechanism of action and efficacy of commonly used modalities. Rheumatol Int.

[14] Quandt SA, Chen H, Grzywacz JG, Bell RA, Lang W, Arcury TA (2005). Use of complementary and alternative medicine by persons with arthritis: results of the National Health Interview Survey. Arthritis Rheum.

[15] Lee MS, Lee MS, Yang CY, Lee SI, Joo MC, Shin BC, Yoo WH, Shin YI (2008). Use of complementary and alternative medicine by rheumatoid arthritis patients in Korea. Clin Rheumatol.

[16] Huang MC, Pai FT, Lin CC, Chang CM, Chang HH, Lee YC, Sun MF, Yen HR (2015). Characteristics of traditional Chinese medicine use in patients with rheumatoid arthritis in Taiwan: a nationwide population-based study. J Ethnopharmacol.

[17] Zhang W, Dai SM (2012). Mechanisms involved in the therapeutic effects of Paeonia lactiflora Pallas in rheumatoid arthritis. Int Immunopharmacol.

[18] Daily JW, Zhang T, Cao S, Park S (2017). Efficacy and Safety of GuiZhi-ShaoYao-ZhiMu Decoction for Treating Rheumatoid Arthritis: A Systematic Review and Meta-Analysis of Randomized Clinical Trials. J Altern Complement Med (New York, NY).

[19] Chen Z, Li XP, Li ZJ, Xu L, Li XM (2013). Reduced hepatotoxicity by total glucosides of paeony in combination treatment with leflunomide and methotrexate for patients with active rheumatoid arthritis. Int Immunopharmacol.

[20] Feng ZT, Xu J, He GC, Cai SJ, Li J, Mei ZG. A systemic review and meta-analysis of the clinical efficacy and safety of total glucosides of peony combined with methotrexate in rheumatoid arthritis. Clin Rheumatol. 2017. 10.1007/s10067-017-3770-y PMC5754451 28748514

[21] Chen YJ, Chang YT, Wang CB, Wu CY (2011). The risk of cancer in patients with rheumatoid arthritis: a nationwide cohort study in Taiwan. Arthritis Rheum.

[22] Kuo CF, Luo SF, See LC, Chou IJ, Chang HC, Yu KH (2013). Rheumatoid arthritis prevalence, incidence, and mortality rates: a nationwide population study in Taiwan. Rheumatol Int.

[23] Shih CC, Liao CC, Su YC, Tsai CC, Lin JG (2012). Gender differences in traditional Chinese medicine use among adults in Taiwan. PLoS One.

[24] Roeters van Lennep JE, Westerveld HT, Erkelens DW, van der Wall EE (2002). Risk factors for coronary heart disease: implications of gender. Cardiovasc Res.

[25] Chen T, Guo ZP, Jiao XY, Jia RZ, Zhang YH, Li JY, Huang XL, Liu HJ (2011). Peoniflorin suppresses tumor necrosis factor-alpha induced chemokine production in human dermal microvascular endothelial cells by blocking nuclear factor-kappaB and ERK pathway. Arch Dermatol Res.

[26] Deng XY, Chen JJ, Li HY, Ma ZQ, Ma SP, Fu Q (2015). Cardioprotective effects of timosaponin B II from Anemarrhenae asphodeloides Bge on isoproterenol-induced myocardial infarction in rats. Chem Biol Interact.

[27] Hang Y, Qin X, Ren T, Cao J (2018). Baicalin reduces blood lipids and inflammation in patients with coronary artery disease and rheumatoid arthritis: a randomized, double-blind, placebo-controlled trial. Lipids Health Dis.

[28] Han C, Guo J (2012). Antibacterial and anti-inflammatory activity of traditional Chinese herb pairs, Angelica sinensis and Sophora flavescens. Inflammation.

[29] Smolen JS, Landewe R, Bijlsma J, Burmester G, Chatzidionysiou K, Dougados M, Nam J, Ramiro S, Voshaar M, van Vollenhoven R (2017). EULAR recommendations for the management of rheumatoid arthritis with synthetic and biological disease-modifying antirheumatic drugs: 2016 update. Ann Rheum Dis.

[30] Tam WY, Chook P, Qiao M, Chan LT, Chan TY, Poon YK, Fung KP, Leung PC, Woo KS (2009). The efficacy and tolerability of adjunctive alternative herbal medicine (Salvia miltiorrhiza and *Pueraria lobata*) on vascular function and structure in coronary patients. J Altern Complement Med (New York, NY).

[31] Ljung L, Askling J, Rantapaa-Dahlqvist S, Jacobsson L (2014). The risk of acute coronary syndrome in rheumatoid arthritis in relation to tumour necrosis factor inhibitors and the risk in the general population: a national cohort study. Arthritis Res Ther.

[32] Bili A, Tang X, Pranesh S, Bozaite R, Morris SJ, Antohe JL, Kirchner HL, Wasko MC (2014). Tumor necrosis factor alpha inhibitor use and decreased risk for incident coronary events in rheumatoid arthritis. Arthritis Care Res.

